# N-Terminal Pro-B-Type Natriuretic Peptide in Risk Stratification of Heart Failure Patients With Implantable Cardioverter-Defibrillator

**DOI:** 10.3389/fcvm.2022.823076

**Published:** 2022-03-01

**Authors:** Yu Deng, Si-Jing Cheng, Wei Hua, Min-Si Cai, Ni-Xiao Zhang, Hong-Xia Niu, Xu-Hua Chen, Min Gu, Chi Cai, Xi Liu, Hao Huang, Shu Zhang

**Affiliations:** Cardiac Arrhythmia Center, Fuwai Hospital, National Center for Cardiovascular Diseases, Chinese Academy of Medical Sciences and Peking Union Medical College, Beijing, China

**Keywords:** N-terminal pro-B-type natriuretic peptide, heart failure, implantable cardioverter-defibrillator, all-cause mortality, appropriate defibrillator shock, restricted cubic spline

## Abstract

**Background:**

The prognostic value of N-terminal pro-B-type natriuretic peptide (NT-proBNP) in heart failure (HF) is well-established. However, whether it could facilitate the risk stratification of HF patients with implantable cardioverter-defibrillator (ICD) is still unclear.

**Objective:**

To determine the associations between baseline NT-proBNP and outcomes of all-cause mortality and first appropriate shock due to sustained ventricular tachycardia/ventricular fibrillation (VT/VF) in ICD recipients.

**Methods and results:**

N-terminal pro-B-type natriuretic peptide was measured before ICD implant in 500 patients (mean age 60.2 ± 12.0 years; 415 (83.0%) men; 231 (46.2%) Non-ischemic dilated cardiomyopathy (DCM); 136 (27.2%) primary prevention). The median NT-proBNP was 854.3 pg/ml (interquartile range [IQR]: 402.0 to 1,817.8 pg/ml). We categorized NT-proBNP levels into quartiles and used a restricted cubic spline to evaluate its nonlinear association with outcomes. The incidence rates of mortality and first appropriate shock were 5.6 and 9.1%, respectively. After adjusting for confounding factors, multivariable Cox regression showed a rise in NT-proBNP was associated with an increased risk of all-cause mortality. Compared with the lowest quartile, the hazard ratios (HRs) with 95% *CI* across increasing quartiles were 1.77 (0.71, 4.43), 3.98 (1.71, 9.25), and 5.90 (2.43, 14.30) for NT-proBNP (*p* for trend < 0.001). A restricted cubic spline demonstrated a similar pattern with an inflection point found at 3,231.4 pg/ml, beyond which the increase in NT-proBNP was not associated with increased mortality (*p* for nonlinearity < 0.001). Fine-Gray regression was used to evaluate the association between NT-proBNP and first appropriate shock accounting for the competing risk of death. In the unadjusted, partial, and fully adjusted analysis, however, no significant association could be found regardless of NT-proBNP as a categorical variable or log-transformed continuous variable (all *p* > 0.05). No nonlinearity was found, either (*p* = 0.666). Interactions between NT-proBNP and predefined factors were not found (all *p* > 0.1).

**Conclusion:**

In HF patients with ICD, the rise in NT-proBNP is independently associated with increased mortality until it reaches the inflection point. However, its association with the first appropriate shock was not found. Patients with higher NT-proBNP levels might derive less benefit from ICD implant.

## Introduction

Sudden cardiac death (SCD) represents a heavy health burden accounting for 15–20% of all deaths around the world (1, 2). Although advances in resuscitation and defibrillation have been made throughout these years, more than 80% of individuals experiencing SCD still could not survive hospital discharge (3, 4). Most SCD events occur in the community-based population without a prior history of structural heart disease, making it difficult to predict (5). Therefore, preventive strategies have been focusing on the high-risk population, such as those with severe heart disease. An implantable cardioverter-defibrillator (ICD) therapy is the widely accepted effective modality to reduce SCD in current guidelines (6, 7). Nevertheless, the selection of patients is mainly based on New York Heart Association (NYHA) functional class and left ventricular ejection fraction (LVEF) (6, 7). A large number of ICD recipients, especially those with Non-ischemic etiology, do not receive appropriate therapy in the long-term follow-up (8–14). Therefore, there is an urgent need to find an additional indicator to identify patients more likely to benefit from ICD therapy.

N-terminal pro-B-type natriuretic peptide (NT-proBNP) is a hormone secreted primarily by the ventricular myocardium in response to increased wall stress due to volume expansion and/or pressure overload in heart failure (HF) patients (15). It is an established biomarker of HF diagnosis and prognosis (15, 16). Moreover, it is recognized as a surrogate indicator for all-cause mortality, HF hospitalization, and HF death (16). In addition, it is associated with myocardial fibrosis (17), which is a well-established arrhythmogenic substrate (18–20). Prior studies have proven that it is associated with an increased risk of SCD both in the general population and patients with heart disease (21–27). This makes it a promising biomarker for risk stratification in patients with ICD. However, because it might increase the occurrence of both SCD and pump failure death, it must be systematically evaluated before it can be applied in the decision-making process of ICD implantation.

The purpose of the present study was to explore the role of NT-proBNP in the risk stratification of HF patients with ICD. To address this hypothesis, we tested its relationship with outcomes of all-cause mortality and first appropriate shock in a population of ischemic or Non-ischemic dilated cardiomyopathy (DCM).

### Study Patients

**Figure 1:**
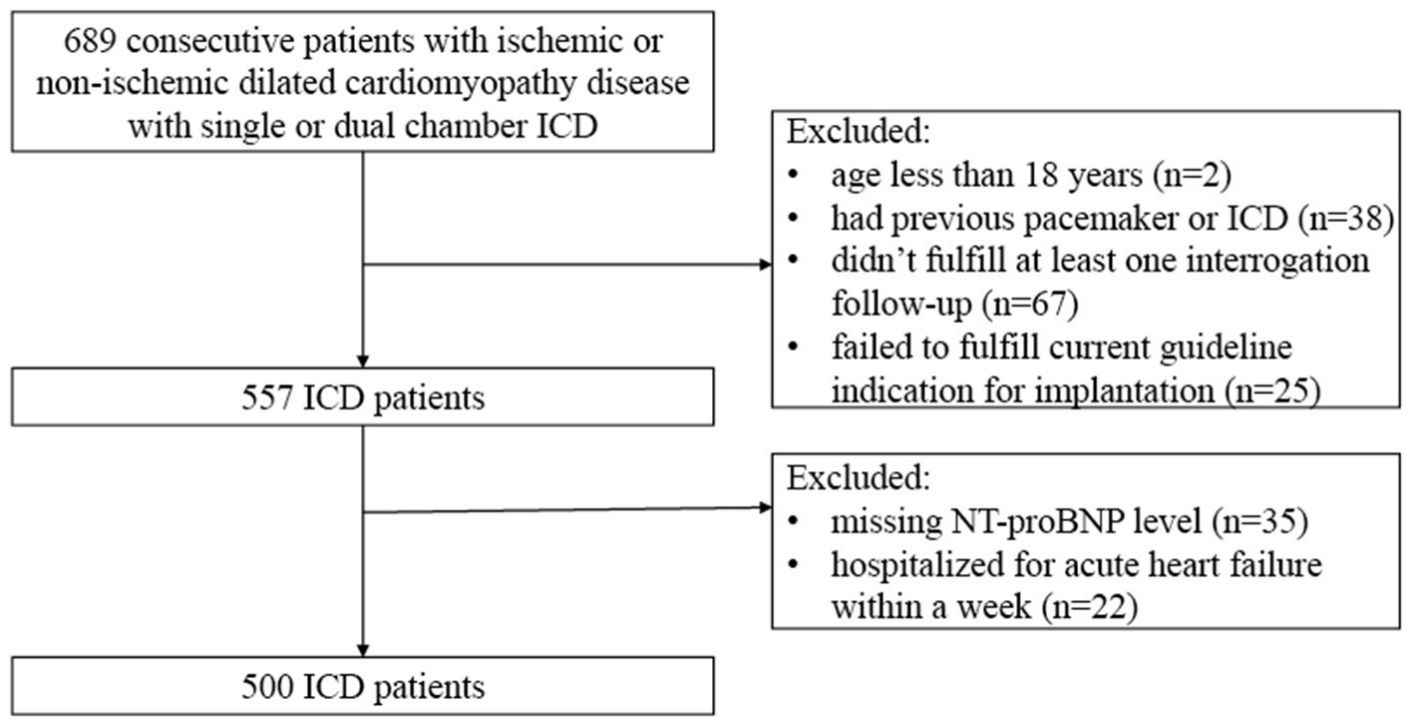
Flowchart on patient inclusion and exclusion.

In total, 689 consecutive patients with ischemic or Non-ischemic dilated cardiomyopathy disease implanted with ICD (single or dual chamber) between January 1, 2013 and September 1, 2020 were enrolled. Ischemic cardiomyopathy (ICM) was defined as left ventricular systolic dysfunction with marked coronary stenosis (28). Non-ischemic DCM was defined as ventricular dilatation and systolic dysfunction in the absence of abnormal loading conditions and marked stenosis (29). The exclusion criteria were (1) age <18 years (*n* = 2), (2) had previous pacemaker or ICD (*n* = 38), (3) did not fulfill at least one interrogation follow-up (*n* = 67), (4) failed to fulfill the current guideline indication for implantation (6, 7) (*n* = 25), (5) had missing NT-proBNP (*n* = 35), and (6) hospitalized for acute HF within a week (*n* = 22). Figure 1 shows the flowchart of the selection of the study population. The study complied with the Declaration of Helsinki and was approved by the Ethics Committee of Fuwai Hospital. All patients gave informed consent.

### Data Collection and Device Programming

Information about demographic characteristics, physical examination, comorbidities, NYHA functional class, and medication history was collected from electronic medical records, which were obtained by trained clinicians at admission. ECGs were obtained by experienced physicians. Blood samples from the participants were taken in the fasting state. NT-proBNP levels were measured within 3 days before ICD implant, using an electrochemiluminescence immunoassay (Roche, Basel, Switzerland) with a limit of quantification (LoQ) of 50 pg/ml by experienced operators.

Although devices were programmed at the discretion of treating physicians, shocks were delivered in the ventricular tachycardia/ventricular fibrillation (VT/VF) zone if the arrhythmia was not terminated by anti-tachycardia pacing or initially applied in the VF zone. Device interrogation results were adjudicated by experienced electrophysiologists. Appropriate therapies were defined as therapies delivered for VT/VF.

### Outcomes

The primary endpoint was all-cause mortality. The survival status was confirmed with medical death records or telephone calls to the patients' relatives or themselves until June 2021. The secondary endpoint was the first appropriate ICD shock. Patients were required to complete device interrogation every 6–12 months or unintended visits after sensing therapies by ICD until June 2021. The dates for the censoring of survival status and interrogation information were not necessarily the same. The appropriate shock was the only type of ICD therapy selected for the secondary endpoint because it was set only to treat the rapid sustained VT or VF (28).

### Statistics

Continuous data are expressed as mean ± SD or the median with the interquartile range (IQR) as appropriate; categorical data are presented as frequencies and percentages. Patients were divided into four groups according to baseline NT-proBNP quartiles. In addition, NT-proBNP was log_10_-transformed for its skewed distribution. Baseline characteristics of the groups were compared with one-way ANOVA for normally distributed continuous variables, the Kruskal–Wallis test for Non-normally distributed continuous variables, and the χ^2^ test for categorical variables. Univariable predictors significant at the *p* < 0.10 level were entered into the subsequent multivariable model. Kaplan–Meier curves were constructed for all-cause mortality, and cumulative incidence curves were constructed for the first appropriate shock. The log-rank test and Fine–Gray test were used to investigate the unadjusted differences of primary and secondary endpoints between groups, respectively. Multivariable Cox proportional hazards models were used to assess the association between NT-proBNP quartiles and all-cause mortality. A Fine–Gray subdistribution hazard model accounting for the competing risk of death was used to assess the association between NT-proBNP quartiles and first appropriate shock. To eliminate the collinearity between LVEF and left ventricular end-diastolic dimension (LVEDD), only LVEF was kept in the multivariable model. The proportional-hazard assumption was assessed with Schoenfeld residuals, and no violations were found. The lowest NT-proBNP quartile was served as the reference group. Tests for trends were calculated by including each corresponding quartile as a continuous numeric variable in the models. Event rates were reported per 100 person-years.

Furthermore, we used a restricted cubic spline with 3 knots according to the Akaike information criterion (AIC) to flexibly model the potential nonlinear effects of NT-proBNP with the outcomes of all-cause mortality and first appropriate shock after adjusting for confounding factors significant in univariable analyses. Nonlinearity was tested by the Wald statistics. If this was detected, we calculated the inflection point by a recursive algorithm to calculate the places where the second derivative of the fitted spline equaled to zero.

Several interactions between NT-proBNP quartiles and baseline characteristics were considered. These included age, gender, body mass index (BMI), primary/secondary prevention indication, ICM/DCM, NYHA functional class, presence of atrial fibrillation (AF), creatinine, and LVEF ( ≤ 35% or >35%). Interactions between variables were considered significant at the value of *p* ≤ 0.1.

Additional sensitivity analyses to evaluate the robustness of our results were also conducted. (1) We replaced LVEF with LVEDD in the multivariable model. (2) We further adjusted for all covariates presented in Table 1 using stepwise selection by AIC rule with the forced entry of NT-proBNP quartiles.

**Table 1:** Clinical characteristics of all patients in terms of baseline NT-proBNP quartiles.

**Characteristics**	**All Patients** **(*n* = 500)**	**Quartile 1** **(*n* = 125)**	**Quartile 2** **(*n* = 125)**	**Quartile 3** **(*n* = 125)**	**Quartile 4** **(*n* = 125)**	***P*-value**
NT-proBNP (pg/mL)	854.2 [402.0; 1,817.8]	219.2 [122.7; 299.5]	625.3 [515.4; 717.3]	1,198.0 [1,029.7; 1,467.0]	3,121.0 [2,296.0; 4,609.7]	<0.001
log-transformed NT-proBNP	2.92 ± 0.49	2.27 ± 0.28	2.78 ± 0.09	3.09 ± 0.10	3.53 ± 0.19	<0.001
Age (years)	60.2 ± 12.0	57.6 ± 12.5	59.3 ± 11.5	60.7 ± 11.9	63.1 ± 11.7	0.003
Male sex	415 (83.0%)	111 (88.8%)	105 (84.0%)	101 (80.8%)	98 (78.4%)	0.146
Non-ischemic etiology	231 (46.2%)	44 (35.2%)	58 (46.4%)	60 (48.0%)	69 (55.2%)	0.016
BMI (kg/m^2^)	25.1 ± 3.5	25.7 ± 3.1	25.9 ± 3.5	24.9 ± 3.4	24.0 ± 3.7	<0.001
Current smoking	263 (52.6%)	74 (59.2%)	72 (57.6%)	63 (50.4%)	54 (43.2%)	0.044
Secondary prevention	364 (72.8%)	97 (77.6%)	97 (77.6%)	89 (71.2%)	81 (64.8%)	0.068
Frequent PVCs	234 (46.8%)	55 (44.0%)	50 (40.0%)	62 (49.6%)	67 (53.6%)	0.143
NSVT	147 (29.4%)	42 (33.6%)	28 (22.4%)	34 (27.2%)	43 (34.4%)	0.121
Dual-chamber ICD	182 (36.4%)	38 (30.4%)	50 (40.0%)	48 (38.4%)	46 (36.8%)	0.412
**NYHA class**						
I/II	295 (59.0%)	95 (76.0%)	85 (68.0%)	69 (55.2%)	46 (36.8%)	<0.001
III/IV	205 (41.0%)	30 (24.0%)	40 (32.0%)	56 (44.8%)	79 (63.2%)	<0.001
**Echocardiogram**						
LVEDD (mm)	64.1 ± 9.25	61.1 ± 8.59	63.2 ± 9.29	65.0 ± 8.74	67.0 ± 9.40	<0.001
LVEF (%)	37.4 ± 11.1	42.8 ± 12.0	40.1 ± 11.8	35.3 ± 8.14	31.3 ± 8.12	<0.001
**Comorbidities**						
AF	136 (27.2%)	19 (15.2%)	26 (20.8%)	37 (29.6%)	54 (43.2%)	<0.001
Hypertension	232 (46.4%)	56 (44.8%)	57 (45.6%)	56 (44.8%)	63 (50.4%)	0.779
Diabetes	120 (24.0%)	27 (21.6%)	24 (19.2%)	27 (21.6%)	42 (33.6%)	0.034
**Laboratory tests**						
Hemoglobin (g/L)	143 ± 18.6	144 ± 14.2	145 ± 15.7	144 ± 21.2	138 ± 21.4	0.008
Creatinine (μmol/L)	97.5 ± 27.8	89.0 ± 21.0	95.4 ± 23.7	95.6 ± 30.9	110 ± 30.1	<0.001
BUN (mmol/L)	7.50 ± 2.95	6.52 ± 2.39	7.19 ± 2.48	7.46 ± 3.19	8.82 ± 3.20	<0.001
Sodium (mmol/L)	140.0 ± 2.5	140.4 ± 2.0	140.0 ± 2.3	139.7 ± 2.7	140.0 ± 2.8	0.168
**Medications**						
ACEI/ARB	383 (76.6%)	95 (76.0%)	101 (80.8%)	91 (72.8%)	96 (76.8%)	0.519
Sacubitril/valsartan	33 (6.60%)	12 (9.60%)	9 (7.20%)	6 (4.80%)	6 (4.80%)	0.360
Beta-blockers	428 (85.6%)	114 (91.2%)	105 (84.0%)	105 (84.0%)	104 (83.2%)	0.232
Amiodarone	297 (59.4%)	68 (54.4%)	76 (60.8%)	81 (64.8%)	72 (57.6%)	0.380
Diuretics	383 (76.6%)	77 (61.6%)	94 (75.2%)	104 (83.2%)	108 (86.4%)	<0.001
MRA	364 (72.8%)	92 (73.6%)	90 (72.0%)	90 (72.0%)	92 (73.6%)	0.984
Digitalis	107 (21.4%)	19 (15.2%)	23 (18.4%)	27 (21.6%)	38 (30.4%)	0.023
Statin	296 (59.2%)	82 (65.6%)	71 (56.8%)	74 (59.2%)	69 (55.2%)	0.355

*Quartile 1: NT-proBNP ≤ 401.9 pg/ml, Quartile 2: 402.0 ≤ 854.2 pg/ml, Quartile 3: 854.3 ≤ 1,817.7 pg/mL, Quartile 4: ≥ 1,817.8 pg/ml*.

*Values are presented as mean ± SD, median, and interquartile range (IQR), or frequency (%)*.

*ACEI/ARB, angiotensin-converting enzyme inhibitor/angiotensin receptor blocker; AF, atrial fibrillation; BMI, body mass index; BUN, blood urea nitrogen; ICD, implantable cardioverter-defibrillator; LVEDD, left ventricular end-diastolic diameter; LVEF, left ventricular ejection fraction; MRA, mineralocorticoid receptor antagonists; NYHA, New York Heart Association; NT-proBNP, N-terminal pro-brain natriuretic peptide; NSVT, Non-sustained ventricular tachycardia; PVC, premature ventricular contractions*.

All analyses were performed using Stata 16.1/IC (StataCorp, College Station, TX) and R 4.1.1 (R Core Development Team, Vienna, Austria), such as the “rm,” “mstat,” “cmprs,” and “survival” packages. A two-sided *p* ≤ 0.05 was considered statistically significant if not otherwise specified.

## Results

Finally, a total of 500 patients were included. The baseline characteristics of patients according to the NT-proBNP quartiles are presented in Table 1. The study population was predominantly male (83.0%). The mean age was 60.2 ± 12.0 years. Median NT-proBNP was 854.3 pg/ml (IQR: 402.0 to 1,817.8 pg/ml). Patients with higher NT-proBNP were more likely to be older, Non-smokers, and have more prevalent DCM, diabetes, and AF (all *p* < 0.05). These patients were more likely to have lower BMI, higher NYHA functional class, lower LVEF, larger LVEDD, higher blood urea nitrogen and creatinine, and receive diuretics and digoxin treatment at baseline (all *p* < 0.05).

**Figure 2:**
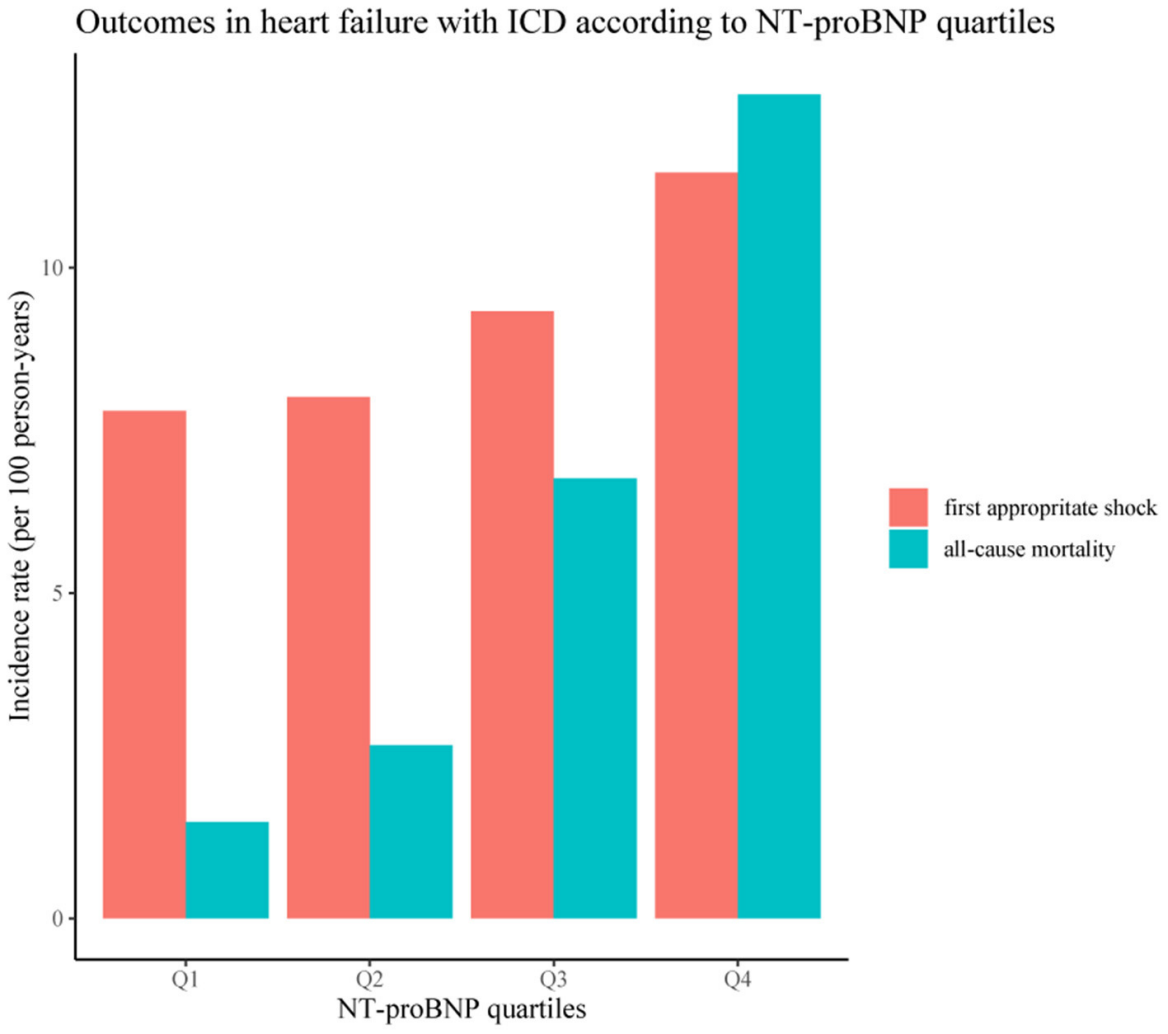
Outcomes in heart failure (HF) with implantable cardioverter-defibrillator (ICD) according to N-terminal pro-brain natriuretic peptide (NT–proBNP) quartiles.

Over a median survival follow-up of 4.1 (IQR 2.8–5.7) years, 106 patients died (incidence 5.61 per 100 person-years; 95% *CI* 4.59–6.78 per 100 person-years). The median interrogation follow-up was 1.7 (IQR 0.8–3.5) years, and 89 patients had their first appropriate shock due to the sustained VT/VF (incidence 9.09 per 100 person-years; 95% *CI* 7.30–11.19 per 100 person-years). The incidence rates of the two outcomes according to NT-proBNP quartiles are shown in Figure 2.

### Relationship Between NT-proBNP and All-Cause Mortality

**Figure 3:**
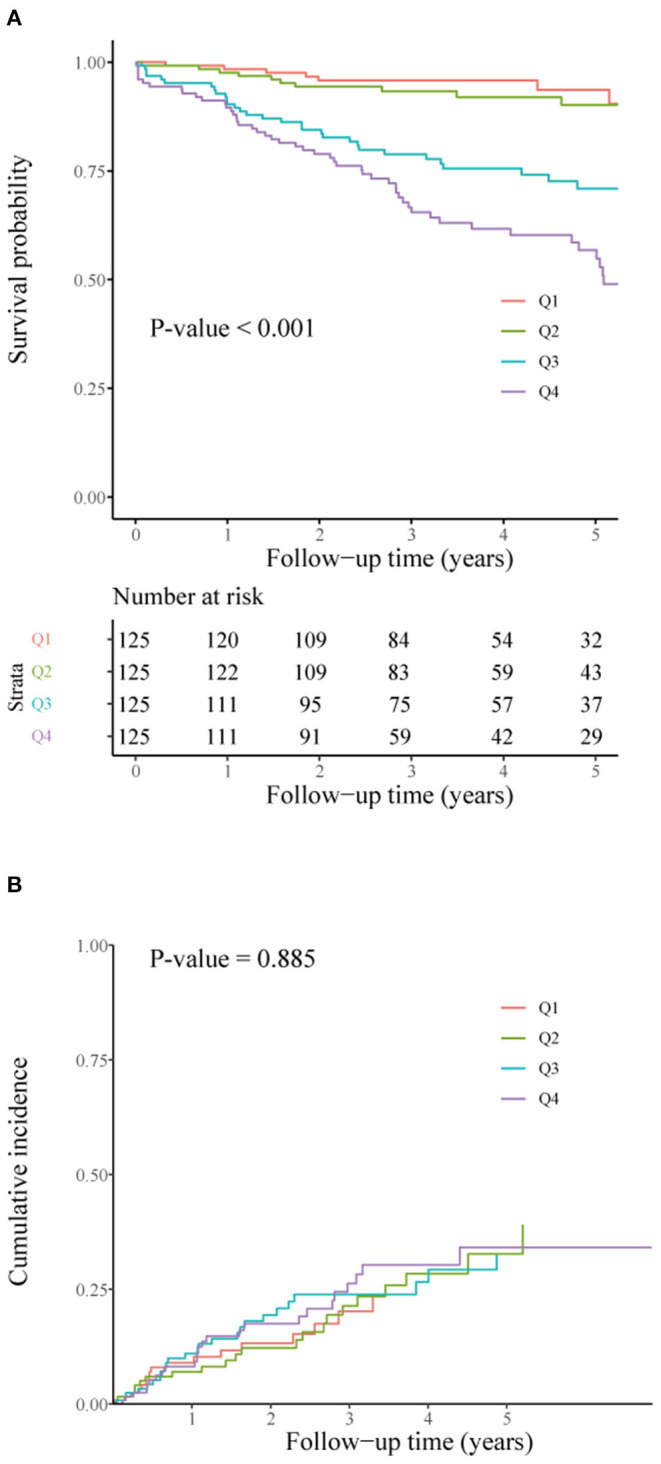
Survival curves for all-cause mortality **(A)** and cumulative incidence curves for first appropriate shock **(B)** according to NT-proBNP quartiles (Q1–Q4). NT-proBNP quartiles were defined as Q1, NT-proBNP ≤ 401.9 pg/ml; Q2, NT-proBNP 402.0 ≤ 854.2 pg/ml; Q3, NT-proBNP 854.3 ≤ 1,817.7 pg/ml; Q4, NT-proBNP ≥ 1,817.8 pg/ml.

Survival curves according to NT-proBNP quartiles are shown in Figure 3A. Patients in the 1st and 2nd quartiles had similar survival (*p* = 0.211), whereas patients in the 3rd and 4th quartiles had significantly worse survival than those in the 1st quartile (*HR* = 4.52, 95% *CI*: 1.99–10.25, *P* < 0.001; *HR* = 8.37, 95% *CI*: 3.80–18.42, *p* < 0.001, respectively). After adjusting for confounding factors, such as age, smoking, prevention indication, ICD/DCM, NYHA functional class, BMI, diabetes, AF, hemoglobin, creatinine, LVEF, and the use of diuretics and digoxin, compared with that in the lowest quartile, the hazard ratios (*HR*s) with 95% *CI* across increasing quartiles were 1.77 (0.71, 4.43), 3.98 (1.71, 9.25), and 5.90 (2.43, 14.30) for NT-proBNP, as shown in Table 2. A similar association was also found after adjusting for LVEDD and further adjusting for other variables in Table 1. The interactions between NT-proBNP and age, gender, BMI, prevention indication, ICM/DCM, NYHA functional class, AF, creatinine, and LVEF were not statistically significant (all *p* > 0.1).

**Table 2:** Unadjusted and adjusted hazard ratios (*HR*s) and subdistribution HRs of outcomes.

**Outcome**	**Events, no**.	**Incidence rate, per 100 person-years**	**Model 1**		Model 2^*^		**Model 3^†^**		**Model 4^‡^**	
			**unadjusted HR/sdHR^#^ (95% CI)**	***P* value**	**adjusted HR/sdHR^#^ (95% CI)**	***P* value**	**adjusted HR/sdHR^#^ (95% CI)**	***P* value**	**fully adjusted HR/sdHR^#^ (95% CI)**	***P* value**
All-cause mortality										
NT-proBNP quartile										
1	7	1.48	1 [Reference]		1 [Reference]		1 [Reference]		1 [Reference]	
2	14	2.66	1.79 (0.72, 4.43)	0.211	1.77 (0.71, 4.43)	0.223	1.77 (0.71, 4.42)	0.221	1.60 (0.64, 3.99)	0.313
3	32	6.76	4.52 (1.99, 10.25)	<0.001	3.98 (1.71, 9.25)	0.001	4.17 (1.80, 9.63)	<0.001	3.99 (1.74, 9.12)	0.001
4	53	12.66	8.37 (3.80, 18.42)	<0.001	5.90 (2.43, 14.30)	<0.001	6.33 (2.64, 15.14)	<0.001	6.61 (2.92, 14.98)	<0.001
*P* for trend^§^				<0.001		<0.001		<0.001		<0.001
*P* for nonlinearity				<0.001		<0.001		<0.001		<0.0001
log10(NT-proBNP)			5.46 (3.46, 8.61)	<0.001	4.16 (2.32, 7.48)	<0.001	4.40 (2.47, 7.82)	<0.001	5.10 (3.03, 8.57)	<0.001
First appropriate shock										
NT-proBNP quartile										
1	17	7.80	1 [Reference]		1 [Reference]		1 [Reference]		1 [Reference]	
2	22	8.01	1.10 (0.58–2.07)	0.769	1.03 (0.54–1.97)	0.938	1.02 (0.53–1.95)	0.952	1.12 (0.59–2.12)	0.738
3	25	9.33	1.23 (0.66–2.29)	0.507	1.10 (0.56–2.14)	0.783	1.14 (0.59–2.19)	0.696	1.28 (0.68–2.43)	0.452
4	25	11.46	1.27 (0.68–2.35)	0.449	1.13 (0.53–2.37)	0.757	1.18 (0.57–2.42)	0.661	1.27 (0.65–2.47)	0.483
*P* for trend^§^				0.404		0.729		0.604		0.434
*P* for nonlinearity				0.751		0.666		0.774		0.771
log10(NT-proBNP)			1.25 (0.80–1.95)	0.334	1.12 (0.64–1.96)	0.688	1.15 (0.67–1.99)	0.608	1.21 (0.74–1.98)	0.443

* *Model 2 was adjusted for age, smoking, prevention indication, ICD/DCM, NYHA class, BMI, diabetes, AF, hemoglobin, creatinine, LVEF, and use of diuretics and digoxin*.

† *Model 3 was adjusted for age, smoking, prevention indication, ICD/DCM, NYHA class, BMI, diabetes, AF, hemoglobin, creatinine, LVEDD, and use of diuretics and digoxin*.

‡ *Model 4 was fully adjusted, such as all variables in model 3 and sex, device type, NSVT, PVC, hypertension, BUN, LVEF, use of ACEI/ARB, sacubitril/valsartan, Beta-blockers, MRA, and use of amiodarone and statin*.

*Stepwise regression by AIC rule with the forced entry of NT-proBNP was used*.

§ *P for linear trend was calculated by including each corresponding quartile as a continuous numeric variable*.

# *The HR and subdistribution HRs were used for the Cox and Fine-Gray models, respectively*.

*sdHR, subdistribution hazard ratio; other abbreviations as in Table 1*.

**Figure 4:**
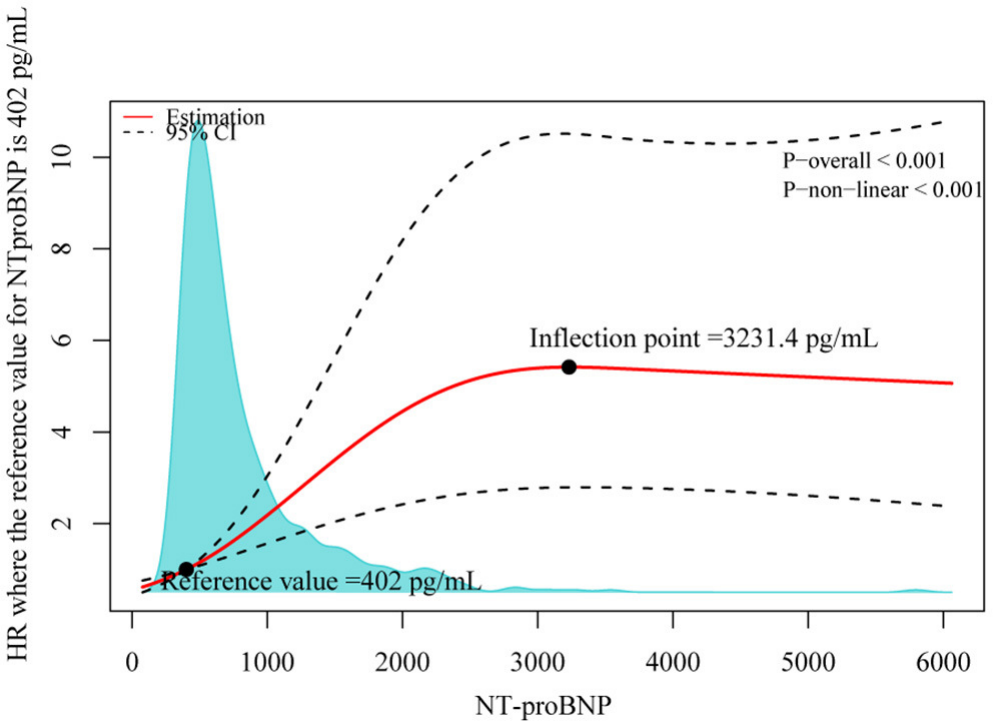
Distributions of NT-proBNP in the overall population and adjusted hazard ratios (*HR*s) of all-cause mortality according to NT-proBNP levels. This plot demonstrates the nonlinear relationship between baseline NT-proBNP levels and the risk of all-cause mortality. A single inflection point was found at 3,231.4 pg/ml. Increases in NT-proBNP from 0 to 3,231.4 pg/ml were associated with a rapid increase in mortality risk but further increases in NT-proBNP >3,231.4 pg/mL were not associated with an increased risk (*p* for nonlinearity < 0.001). The dotted line indicates the corresponding 95% *CI*s. The 25th percentile of NT-proBNP (402.0 pg/ml) was set as a reference. A density plot is also drawn to show the distribution of NT-proBNP.

The restricted cubic spline shown in Figure 4 displays the association between NT-proBNP and all-cause mortality (*p* for nonlinearity < 0.001). The risk of all-cause mortality increased rapidly until it reached the inflection point, which was equal to 3,231.4 pg/ml. Above this point, the curve was relatively flat, which meant that the risk would not increase afterward.

### Relationship Between NT-proBNP and First Appropriate Shock

Cumulative incidence curves according to NT-proBNP quartiles in Figure 3B did not show a difference in time to the first appropriate shock (*p* = 0.885). In the multivariable competing risk analyses shown in Table 2, NT-proBNP, regardless of whether it was coded as a categorical variable or log-transformed as a continuous variable, did not show a significant association with the first appropriate shock after adjusting for variables significant in univariable analyses (all *p* > 0.05). Even after further adjusting for other variables using the AIC rule, it was not significant (*p* > 0.05). Additionally, we examined the potential nonlinear association between NT-proBNP and first appropriate shock using the restricted cubic spline, while no such association was found (*p* for nonlinearity = 0.666). The interactions between NT-proBNP and predefined factors were also not significant (all *p* > 0.1). Sensitivity analyses by adjusting LVEDD showed similar results. Instead, LVEDD itself was found to be a significant predictor (per 5 mm increase, subdistribution HR = 1.13, 95% *CI:* 1.01–1.26, P = 0.035). Overall, we did not observe the association between NT-proBNP levels and the first appropriate shock.

## Discussion

The prognostic importance of NT-proBNP has been broadly studied in patients with HF, but remains largely unexplored in HF patients with ICD. In our study, we found that patients with higher NT-proBNP levels had a lower survival probability, however, did not have a higher risk of appropriate shock. Therefore, these patients might derive less benefit from an ICD implant. Our study validated the prognostic importance of NT-proBNP associated with all-cause mortality in previous studies. Nonetheless, our findings raised key questions about the utility of NT-proBNP in the risk stratification of SCD.

According to current guidelines (6, 7), a lot of HF patients implanted with ICDs would not receive appropriate ICD shock in the long-term follow-up (8–11). Consequently, there is an urgent need to find a new risk stratification marker in addition to LVEF and NYHA. Since published data have shown that NT-proBNP has a close relationship with all-cause mortality (15, 16), pump failure death (15, 16), and sudden death in a variety of populations (21–27), it is also expected to be a promising marker for HF with ICD.

As expected, we demonstrated that NT-proBNP conferred an increased risk of all-cause mortality. Nonetheless, particular attention must be paid that our population was comprised of patients with ICD, in which death due to cardiac arrest was greatly prevented (6, 7). Therefore, it could be speculated that the predominant modes of death were pump failure in our setting. In this regard, our finding was consistent with previous studies showing that higher NT-proBNP was associated with an increased risk of HF death (24, 25, 27, 30). To the best of our knowledge, our study is the first to characterize NT-proBNP levels with all-cause mortality using a smooth spline in patients with ICD patients. The spline illustrated the relationship between NT-proBNP and all-cause mortality as a logarithmic curve. This was also in line with our finding that log-transformed NT-proBNP was a significant predictor in the multivariable models. Therefore, it justifies the convention that NT-proBNP should be log-transformed in the data analysis process (18, 22, 24, 27). Furthermore, our results showed that once the NT-proBNP level surpassed the inflection point, the risk of all-cause mortality would not increase further. This might reflect the ceiling effect of NT-proBNP. Unfortunately, most previous studies failed to find this effect (24, 25, 27, 30). Of note, our inflection point might not be suitable for other populations. Nonetheless, it shows a phenomenon that an extremely high NT-proBNP value does not necessarily translate into an extremely high risk of death. This might be explained by the fact that even when the NT-proBNP level is high, it could be considerably reduced when treatments are further intensified in stable patients (15, 31–33). However, we cannot rule out that this finding represented the play of chance. It needs to be replicated in the future.

In contrast with published studies, we failed to demonstrate the connection between NT-proBNP and SCD, which was substituted by appropriate shock in our study. In fact, according to the definition of SCD (34), precise adjudication of SCD was almost impossible except for evidence found at autopsy. In this regard, the endpoint we used might be more accurate to reflect the actual rate of sudden death from a cardiac cause. On the other hand, since NT-proBNP is a surrogate for intracardiac volumes and filling pressures (15, 20, 35), echocardiographic parameters might reflect this nature more directly. Among these, LVEDD was proved to have a positive relationship with increased intracardiac pressures and also to be positively correlated with NT-proBNP (36). A case-cohort study of 418 patients with SCD and 329 controls based on the general population suggested that moderate or severe left ventricular dilation was an independent predictor of SCD (37). Given the strong relationship between NT-proBNP and LVEDD, inference can only be considered robust when two variables are put together in the multivariable model. Otherwise, it might lead to a biased result. However, most studies failed to adjust for LVEDD in their analyses (22, 23, 26, 27, 38). Furthermore, although NT-proBNP showed significant associations with SCD (22, 38), a cause-and-effect relationship might not exist. A variety of studies have demonstrated that NT-proBNP has a stronger relationship with all-cause mortality and pump failure death than SCD (23–25, 27) by showing a higher *HR*. Clinical models, including NT-proBNP, to predict pump failure death also showed better discrimination ability than to predict SCD (24, 38). These findings indicate that NT-proBNP might not have a direct effect on SCD. Conversely, it might be just a marker of HF progression (39). As a result, the Danish study to assess the efficacy of ICDs in patients with non-ischemic systolic heart failure on mortality (DANISH) trial found that only patients in the subgroup of NT-proBNP <1,177 pg/ml had an increased benefit of ICD implant (10). Instead, we found that LVEDD was a predictor of SCD, consistent with a previous meta-analysis that included four relevant studies (40). This finding further indicates that NT-proBNP might not be a proper predictor for SCD. In conclusion, a single NT-proBNP level should not be used as a risk stratification tool for SCD.

Our finding was contrary to an analysis of 342 patients with primary prevention ICD after a median follow-up of 35 months (20). The authors found that NT-proBNP was not associated with its combined outcomes including death from any cause while it was positively associated with appropriate ICD therapies. An earlier study also revealed that NT-proBNP was associated with both appropriate ICD therapies and total mortality (19). In contrast, our finding was consistent with the risk prediction model developed by Bergau et al. (41), in which NT-proBNP was a predictor of all-cause mortality, while it was not a predictor of ICD shock (42). Disparities between these studies might be explained by their population, conduction, and slightly different definitions of endpoints. Most importantly, these studies failed to handle the Non-normality of NT-proBNP properly, where the first two simply dichotomized it while the third treated it as a continuous normal distribution variable. In this regard, their conclusions were less reliable than ours.

Our study has some limitations. First, the mean follow-up duration of shock status was less than that of survival status. It might undermine the power of our analysis. However, it is comparable with other studies (19, 23) dedicated to solving this hypothesis. Moreover, the follow-up period does not have an influence on the *HR* in the proportional hazards model in the absence of time-varying variables (43). Second, we only explored the baseline effect of NT-proBNP instead of repetitive levels. Dynamic changes in NT-proBNP levels and echocardiography parameters might provide incremental information on prognosis (30–33, 44). For example, an improvement in LVEF was associated with reduced ICD therapy and lower mortality (44). However, due to the retrospective nature of our study, it is hard to strictly choose unified timepoints to define serial change. Nonetheless, our study demonstrated that a single baseline NT-proBNP level was a predictor of death, which is easier to interpret and use in clinical setting. Third, our endpoint did not include anti-tachycardic pacing, which might also be triggered by fatal arrhythmic events. In fact, the inclusion of anti-tachycardic pacing is not proper because it was mainly designed for treating hemodynamically stable, slower rate ventricular tachyarrhythmia. As a result, only appropriate shock was included as the endpoint.

## Conclusion

We conducted a thorough exploration of the association of NT-proBNP with all-cause mortality as well as the first appropriate shock by restricted cubic spline analysis. We found increasing NT-proBNP levels were related to an increased risk of death with a ceiling effect at 3,231.4 pg/ml, but not related to the first appropriate shock. Therefore, patients with higher NT-proBNP might derive less benefit from ICD implant. It still needs further investigation to confirm our results.

## Data Availability Statement

The original contributions presented in the study are included in the article/supplementary material, further inquiries can be directed to the corresponding author.

## Ethics Statement

The studies involving human participants were reviewed and approved by Ethics Committee of Fuwai Hospital. The patients/participants provided their written informed consent to participate in this study.

## Author Contributions

WH, YD, SZ, H-XN, X-HC, MG, and CC contributed to conception and design of the study. YD, N-XZ, XL, M-SC, S-JC, and HH organized the database. YD and M-SC performed the statistical analysis. YD wrote the first draft of the manuscript. WH revised the manuscript. All authors contributed to manuscript revision, read, and approved the submitted version.

## Conflict of Interest

The authors declare that the research was conducted in the absence of any commercial or financial relationships that could be construed as a potential conflict of interest.

## Publisher's Note

All claims expressed in this article are solely those of the authors and do not necessarily represent those of their affiliated organizations, or those of the publisher, the editors and the reviewers. Any product that may be evaluated in this article, or claim that may be made by its manufacturer, is not guaranteed or endorsed by the publisher.
